# Does the African Citrus psyllid, *Trioza erytreae* (Del Guercio) (Hemiptera: Triozidae), Represent a Phytosanitary Threat to the Citrus Industry in Mexico?

**DOI:** 10.3390/insects12050450

**Published:** 2021-05-14

**Authors:** Saúl Espinosa-Zaragoza, Nidia Bélgica Pérez-De la O, Juan Francisco Aguirre-Medina, Víctor López-Martínez

**Affiliations:** 1 Facultad de Ciencias Agrícolas, Campus IV, Universidad Autónoma de Chiapas, Entronque Carretera Costera y Pueblo de Huehuetán, Huehuetán 30660, Chiapas, Mexico; saulez1@gmail.com (S.E.-Z.); nidia.perez@unach.mx (N.B.P.-D.l.O.); juan.aguirre@unach.mx (J.F.A.-M.); 2 Facultad de Ciencias Agropecuarias, Universidad Autónoma del Estado de Morelos, Av. Universidad 1001, Col. Chamilpa, Cuernavaca 62209, Morelos, Mexico

**Keywords:** ecological niche model, environmental availability, environmental connectivity, invasive species

## Abstract

**Simple Summary:**

The African citrus psyllid, *Trioza erytreae* (Del Guercio) (Hemiptera: Triozidae) is an invasive species for citrus crops. In its native range is the main vector of *Candidatus* Liberibacter africanus (CLaf), a pathogen that causes huanglongbing (HLB). For Mexico, *T. erytreae* could threat the citrus industry in a potential invasion but until now, the best chances to prevent its damage is analyzing if the country has the ecological conditions suitable for this psyllid. In this study we used the ecological niche modeling approach to explore which areas in Mexico has the environmental suitability for the *T. erytreae* establishment. Additionally, the potential role of an alternate host, *Casimiroa edulis* La Llave (Rutaceae), and five points of entry into the country, in the potential *T. erytreae* dispersion were analyzed. Mexico citrus areas has a wide environmental suitability for *T. erytreae*, including the main federal entity (Veracruz). The natural distribution of *C. edulis* matches with the *T. erytreae* environmental suitability and citrus areas, and could expand its distribution across the country. For preventive monitoring strategies, the port of Veracruz is a vital point for phytosanitary agencies, because of its proximity to citrus areas.

**Abstract:**

The African citrus psyllid, *Trioza erytreae* (Del Guercio) (Hemiptera: Triozidae), is a vector of *Candidatus* Liberibacter africanus (CLaf), a pathogen that causes huanglongbing (HLB) in Africa. *Trioza erytreae* has invaded areas of Asia and Europe and has threatened citrus production due to its biological habits and the transmission of CLaf. Mexico is a country where citrus production has a vital role from the economic and social point of view. Therefore, ecological niche modeling (ENM) was used to determine if Mexico has the environmental availability that will allow *T. erytreae* invasion. We analyzed whether or not the distribution of *Casimiroa edulis* La Llave (Rutaceae) in the country could be a factor that enables the dispersal of *T. eytreae*. The environmental connectivity between five points of entry into the country (two ports and three airports) was explored to determine possible routes of dispersal of *T. erytrae*. The results showed that Mexico has wide availability for the invasion of the African citrus psyllid, which coincides with essential citrus areas of the country and with the distribution of *C. edulis*. Of the entry points studied, the Port of Veracruz showed nearby areas with environmental connectivity. Preventive monitoring measures for *T. erytreae* in Mexico should focus on Veracruz state because it has an entry point, ideal environmental availability, citrus areas, and specimens of *C. edulis*.

## 1. Introduction

Huanglongbing (HLB) or citrus greening is one of the most destructive diseases in the citrus industry [1,2]. It is mainly associated with the transmission of *Candidatus* Liberibacter africanus (CLaf), *Candidatus* Liberibacter americanus (CLam), and *Candidatus* Liberibacter asiaticus (CLas) [2,3]. The vectors of these Gram-negative, non-cultivable bacterial strains are the Asian citrus psyllid, *Diaphorina citri* Kuwayama (for CLam and CLas), and the African citrus psyllid, *Trioza erytreae* (Del Guercio) for CLaf [4].

*Diaphorina citri* has moved from Asia to spread around the world [5]. It has established and causes severe damage to the citrus industry, affecting quality and yield in Mexico [6], Brazil [7], USA [8], and other countries [9]. *Trioza erytreae* is an Afrotropical species, where it feeds on commercial and backyard citruses, such as lemon [*Citrus limon* (L.) Burm. F.], sweet orange (*Citrus sinensis* (L.) Osb.), and tangerine (*C. reticulata* Blanco) [10,11,12,13,14]. It also feeds on crops of *Casimiroa edulis* La Llave [15], and local wild Rutaceae [16], although in the latter, there is no clear association in infection with CLaf [17].

Recently, this species expanded its distribution to islands around Africa, Asia (Arabian Peninsula), and Europe [10,18,19,20,21]. Its ability to transmit CLaf, and even CLas, to European citruses is worrying [20]. Infested trees show leaves with structures resembling open galls [22], spontaneous defoliation, and wilt. When a tree is infected with CLaf, fruit drop, descending death, and general weakening of the tree are reported [23].

The ecological adaptations of the vector and the expression of the disease have determined its distribution. In essence, it seems that CLaf prefers citruses growing at heights above 700 masl, relatively warm, and cold sites, but CLas is found in warmer lowlands [3,24]. This pattern coincides with the preferences of *T. erytreae*: high altitudes, with colder and more humid climates [20,25,26]. Contrastingly, *D. citri* tolerates extreme temperatures [27] and is sensitive to lowland rain.

*Trioza erytreae* successfully broke through each of the invasion barriers (geographic, survival, spreading) to reach the impact stage for an invasive species [28,29]. With its recent intrusion of Europe, it seems a matter of time and transport route for *T. erytreae* to reach America, where CLaf does not exist. In Mexico, citrus fruits are grown commercially in 602,309.59 hectares, with a production volume of 8,375,803.58 tons in 2018 [30]. They are grown in many ecological conditions and currently face severe impact from *D. citri* and CLas transmission [6,31]. Additionally, white sapote, *C. edulis*, is a species whose domestication center is the central zone of Mexico, where it grows wild and backyards in subtropical areas at altitudes between 1500–2500 masl [32].

This allows us to ask the following questions: Does Mexico offer environmental availability for possible colonization and invasion by populations of *T. erytreae*? If this is true, which citrus areas are threatened? Will the distribution of *C. edulis* facilitate the spread of the African citrus psyllid in the country? Do entry points to Mexico have the environmental conditions for *T. erytreae* survival?

To answer these questions, the aims of this study were to calculate the ecological niche [33] of the African citrus psyllid, and we projected it onto Mexico. Subsequently, with the circuit theory [34], we evaluated the connectivity between entry points to the country, citrus production areas, and spots with the presence of *C. edulis*. The purpose of the information is to guide decision-making at monitoring and sampling sites for preventive detection of *T. erytreae* in Mexico. Ecological Niche Modeling (ENM) is widely used to calculate areas environmentally suitable for invasive species in current or future scenarios at local, regional, or global geographical levels [35,36,37]. It is based on the use of species distribution points, analysis of the influence of environmental factors in the design of the ecological niche and the projection in the geographical plane. The circuit theory is applied to predict the spread of invasive species, evaluate landscape connectivity, and calculate individuals’ flow direction [38,39]. Maps derived from this approach are used by national plant and animal health protection organizations [40] predicting the invasion site successfully and tracing dispersion patterns [41,42,43,44].

Predictive studies help to better prioritize management strategies by estimating the potential invasive range of invasive species [45]. However, predicting the full potential invasive range of an invasive species may not be as relevant as accurately predicting the areas that are most likely to be colonized [45]. Mapping routes with ecological connectivity can decrease the impact of *T. erytreae* if it reached Mexico, and could delay or impede spreading throughout the citrus-producing regions of the country. A similar analysis was applied for the Asian long-horned beetle, *Anoplophora glabripennis* Motschulsky (Coleoptera: Cerambycidae), an invasive species for North America, and allowed to improve the most efficient implementation of resources and detection samples [39].

## 2. Materials and Methods

### 2.1. Biological Information and Delimitation of the Study Area

**Figure 1:**
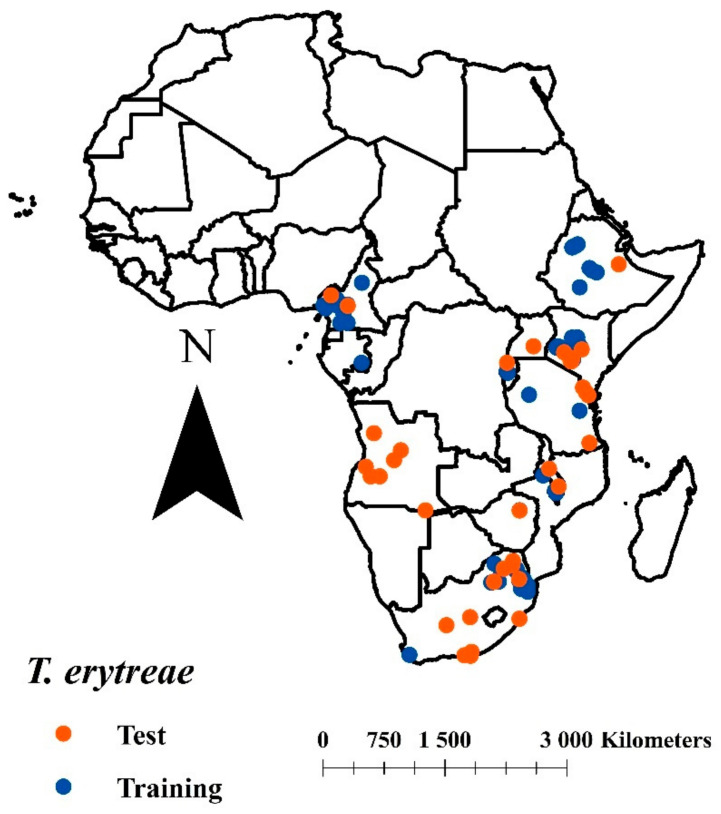
Distributional data of *Trioza erytreae* used training and model evaluation.

A database was built with *T. erytreae* distribution points obtained from scientific literature and diversity databases (Global Biodiversity Information Facility, GBIF; Natural History Museum, NHM, London, UK), with a total of 267 points. Subsequently, duplicate points were eliminated, and to decrease effects from spatial correlation, locations near a radius of 5 km were removed. The final points were divided as follows: 71 points for training and 51 for evaluation [Figure 1].

Additionally, 486 *C. edulis* distribution points were downloaded from the GBIF and SEINet diversity portals.

### 2.2. Model Calibration

Calibrated models based on species accessibility, often reflect true ecological niches [33] more accurately. The *T. erytreae* accessibility area was established according to the BAM diagram [46], where “B”: variables related to the population that include limited food resources, presence of competitors and predators (however, “B” is usually omitted due to the complexity of the final temporal space resolution that is needed); “A”: stipulates ranges or values of environmental variables where a species can survive, for which 19 bioclimatic variables were downloaded from WorldClim v 2.0 [47]; and, “M”: the accessible areas of the species during relevant periods [33,48], for which the native area of *T. erytreae* was delimited using worldwide ecoregions [49].

The information was worked in Rstudio^®^ ver. 3.3 [50] and the Kuenm package [51]. Kuenm calibrates the ecological niche model in two steps: first, creation of a large number of candidate models [developed with Maxent with different parameterization: Linear (L), Quadratic (Q), Product (P), Threshold (T), and Hinge (H), and different the regularization multiplier parameter values]; and second, evaluation and selection of the best models [51]. Different Multiplier regularization values (0.5, 1, 2, 3) were applied to explore those that balance the fit and complexity of the models [52]. Combinations of five Maxent features (Linear, Quadratic, Product, Threshold, and Hinge) and four values of Regularization multiplier (0.1, 0.5, 1, 2, and 3) produced a total of 231 models.

The candidate models were evaluated with Kuenm to filter out those that are statistically significant (*p* = 0.05). Then the accuracy of the models was evaluated with the area under the curve (AUC index) and classified under the following scale: 0.9–1 = excellent; 0.8–0.9 = good; 0.7–0.8 = fair; 0.6–0.7 = poor; and 0.5–0.6 = fail [53]. The omission rate criterion was applied to the resulting models. Finally, among the significant and low omission candidate models, those with Delta AICc values below two were selected [51]. Once the best model was selected according to the filtration of the evaluation metrics, the transfer from the calibration area (Africa) to the study area (Mexico) was carried out with Kuenm. The projections to other geographic areas are mainly an expression of the abiotic niche, combinations of environmental factors based on model estimates that are similar to the areas where the species occurs [54].

To estimate the susceptibility to the *T. erytrae* invasion, each citrus-producing federal entity was classified according to the territorial proportion that presents environmental availability for the psyllid as follows: high risk when the state showed more than 60% of its territory with adequate environmental availability, medium risk when 30–60% of the surface showed availability, and low risk when less than 30% was environmentally suitable for the psyllid. To analyze the potential impact of the *T. erytreae* invasion the citrus-producing states were classified by cultivated area [30] as follows: important states (with a cultivated area greater than 10,000 hectares), states with medium importance (an area greater than a thousand, but less than 10,000 hectares), and of little importance (states with less than a thousand hectares). For the present analysis, lemon, orange, tangerine, and grapefruit were considered (Table 1).

A MESS (Multivariate Environmental Similarity Surfaces) analysis was done in Maxent ver. 3.4.0 [55] to compare the values of the climatic variables in the study area with respect to the distribution of the values from the reference points, and identify where a problem may exist [56]. Negative values indicate different levels of dissimilarity and point to the variable that drives the value of the multivariate environmental similarity surface in each cell of the grid [57].

### 2.3. Conductance Area Model

The bioclimatic connectivity was estimated using the Circuite Scape version 4.0.5 software [34]. One of the tools of this program is to predict spreading routes. It uses raster maps, where each of the grids represents the characteristics of the landscape. In the process, each grid is replaced by a node (connecting points that can represent habitat patches, populations, or cells in a raster landscape) and connects to its neighbors at an edge; the edges represent the weight proportional to the number of individuals that move [58,59]. Bioclimatic connectivity is based on circuit theory, which can be applied to predict probabilities of successful spreading, measure ecological connectivity, generate connectivity measures, and identify critical connective elements [34].

The ecological niche model calculated for *T. erytreae* was used as a raster for the analysis of bioclimatic connectivity, to find the best possible route between areas cultivated with citruses in Mexico through conductance. The latter is related to the probability that a walker chooses to move through a cell or along the edge of a graph in relation to other available cells [34]. For the present model, 71 geo-referenced points from the states of Veracruz, Michoacan, Quintana Roo, Puebla, Hidalgo, Tabasco, and Oaxaca were used. These were selected because they reported surfaces cultivated with orange or lemon and because they presented environmental availability for *T. erytreae*, according to the previous ecological niche modeling. As entry points, the ports of Lázaro Cárdenas and Veracruz were included, as well as the airports of Mexico City, Monterrey, and Cancun.

## 3. Results

### 3.1. Environmental Availability for Trioza erytreae in Mexico

**Figure 2:**
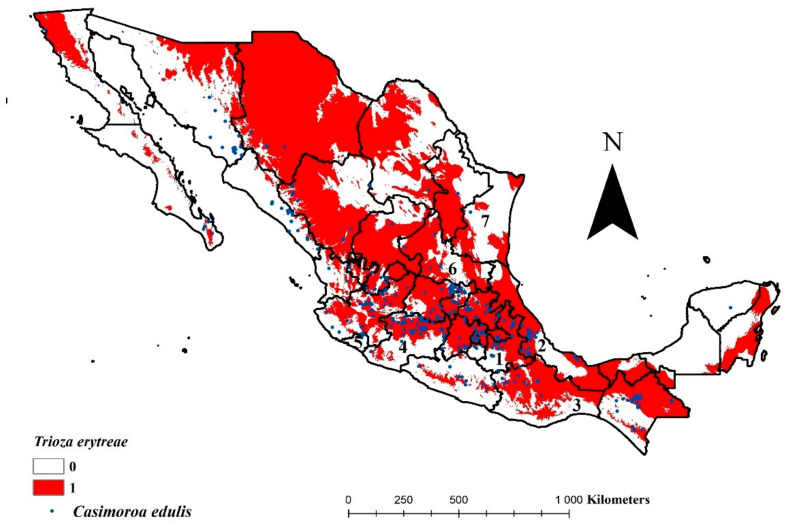
Environmental suitability of the African citrus psyllid, *Trioza erytreae* (Hemiptera: Triozidae) in Mexico, according to division states. White= areas without environmental suitability; red= areas with environmental suitability. State identifications: (1) Aguascalientes, (2) Baja California, (3) Baja California Sur, (4) Campeche, (5) Coahuila, (6) Colima, (7) Chiapas, (8) Chihuahua, (9) Ciudad de Mexico, (10) Durango, (11) Guanajuato, (12) Guerrero, (13) Hidalgo, (14) Jalisco, (15) Estado de Mexico, (16) Michoacan, (17) Morelos, (18) Nayarit, (19) Nuevo Leon, (20) Oaxaca, (21) Puebla, (22) Queretaro, (23) Quintana Roo, (24) San Luis Potosi, (25) Sinaloa, (26) Sonora, (27) Tabasco, (28) Tamaulipas, (29) Tlaxcala, (30) Veracruz, (31) Yucatan, and (32) Zacatecas.

The AUC value for the model selected for *T. erytreae* was 0.830; the accuracy for predictive occurrence is considered good. Seven bioclimatic variables determined the environmental suitability of *T. erytreae* in Mexico (mean temperature of wettest quarter, precipitation of driest month, precipitation of warmest quarter, isothermality, mean diurnal range, precipitation seasonality, and, annual mean temperature) (Table 2). Still, two of them explained 94.3% of the variation within the model (mean temperature of wettest quarter with 69%, and precipitation of driest month with 25.3%). The niche model calculated for *T. erytreae* covers most of the country; medium environmental availability was projected for Baja California, Baja California Sur, Sonora, Guerrero, and Tamaulipas (Figure 2). Minimal or no environmental availability was observed in Campeche, Morelos, Sinaloa, and Yucatan. The rest of the federal entities show broad areas where the ecological niche of *T. erytreae* coincides. The Mexican Pacific coast did not show environmental availability, except to the northwest of Baja California. In contrast, the Gulf of Mexico coast showed greater availability, mainly in Tamaulipas, Veracruz, and Tabasco.

### 3.2. Potentially Affected Citrus-Producing Area

50% of the citrus-producing states with more than 10 thousand hectares showed a high risk of being invaded by *T. erytreae* (Table 3). Veracruz, San Luis Potosi, Puebla, Oaxaca, and Tabasco stand out for their importance; these states represent 59.4% of the surface of Mexico dedicated to citrus production. Michoacan, Tamaulipas, and Nuevo Leon showed medium risk, while Colima and Yucatan are at low risk. States with areas of less than 10,000 and more than 1000 hectares generally showed medium to low risk (Table 2), with a total of 20,117.99 hectares (5.1% of the national total. Jalisco and Hidalgo were classified as high risk (13,299.73 ha). For entities with smaller citrus areas (less than a thousand hectares), the situation was contrary to the previous group, 1674.6 hectares were categorized with a high level of risk (Table 2); however, they represent only 0.28% of the national total. Morelos and Baja California showed low risk.

### 3.3. MESS Analysis

**Figure 3:**
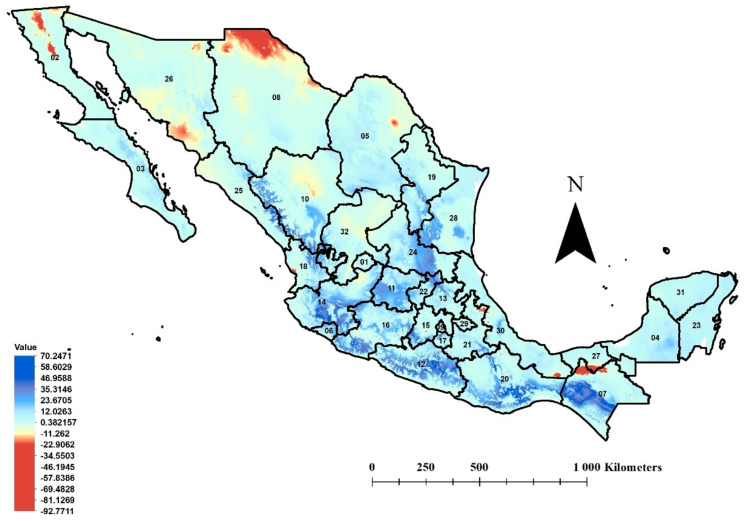
MESS analysis in Mexico for *Trioza erytreae*. Dark blue color and positive values indicate sites with climatic conditions similar to those in the insect species’ native ecological niche; dark red color and negative values indicate degrees of dissimilarity. State identifications: (1) Aguascalientes, (2) Baja California, (3) Baja California Sur, (4) Campeche, (5) Coahuila, (6) Colima, (7) Chiapas, (8) Chihuahua, (9) Ciudad de Mexico, (10) Durango, (11) Guanajuato, (12) Guerrero, (13) Hidalgo, (14) Jalisco, (15) Estado de Mexico, (16) Michoacan, (17) Morelos, (18) Nayarit, (19) Nuevo Leon, (20) Oaxaca, (21) Puebla, (22) Queretaro, (23) Quintana Roo, (24) San Luis Potosi, (25) Sinaloa, (26) Sonora, (27) Tabasco, (28) Tamaulipas, (29) Tlaxcala, (30) Veracruz, (31) Yucatan, and (32) Zacatecas.

The areas with the highest *T. erytreae* environmental similarity between the projected niche in the study area and the native niche were registered far from the country’s coastlines, with a tendency towards the inland (Figure 3): in the limits between Sinaloa and Durango, the northeast of Nayarit, the south central zone of Jalisco, south of Guanajuato, center of San Luis Potosi, southeastern and south of Tamaulipas, south and center of Michoacan, most of Guerrero, in strips from east to west in Oaxaca and Chiapas, and in isolated areas of Veracruz, Campeche, and Nuevo León. The greatest dissimilarity of the ecological niche was projected for the north of Chihuahua, south of Sonora, north-central Baja California, the limits of Tabasco-Chiapas, and small areas in Coahuila, Veracruz, Nayarit, Puebla, and the Estado de Mexico.

### 3.4. Environmental Connectivity

**Figure 4:**
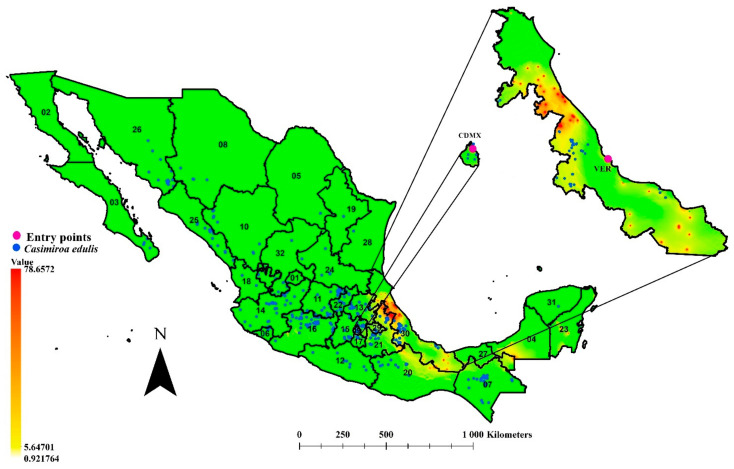
Environmental connectivity for *Trioza erytreae* and two entry points to Mexico: the International Benito Juarez Airport (CDMX) and the Veracruz Port (VER). Blue points show the distributional records of *Casimiroa edulis* in Mexico, an alternative host for *T. erytreae*. Dark red color and highest values indicate areas with environmental connectivity, yellow color and lower values indicate reduced degrees of connectivity; green color indicate not connectivity. State identifications: (1) Aguascalientes, (2) Baja California, (3) Baja California Sur, (4) Campeche, (5) Coahuila, (6) Colima, (7) Chiapas, (8) Chihuahua, (9) Ciudad de Mexico, (10) Durango, (11) Guanajuato, (12) Guerrero, (13) Hidalgo, (14) Jalisco, (15) Estado de Mexico, (16) Michoacan, (17) Morelos, (18) Nayarit, (19) Nuevo Leon, (20) Oaxaca, (21) Puebla, (22) Queretaro, (23) Quintana Roo, (24) San Luis Potosi, (25) Sinaloa, (26) Sonora, (27) Tabasco, (28) Tamaulipas, (29) Tlaxcala, (30) Veracruz, (31) Yucatan, and (32) Zacatecas.

It was not possible to calculate the environmental connectivity between the plotted points of entry to Mexico and the points with citrus fruits analyzed in Michoacan, Morelos, Hidalgo, southern Puebla, and Quintana Roo. The last would mean a decrease in the risk of spreading from airports (Mexico City and Monterrey) and ports (Lazaro Cardenas, Cancun) towards fruit growing areas. However, citrus areas northwest of the Port of Veracruz showed environmental connectivity (Figure 4); here is the most crucial citrus-producing region of Veracruz (municipality of Martinez de la Torre). The connectivity extended to the north of Puebla and southeast in the Veracruz-Puebla limits, although with decreased connectivity.

### 3.5. Role of Casimiroa edulis in the Potential Spreading of T. erytreae to Citrus-Producing Areas

The ecological niche model of *T. erytreae* showed great coincidence with the *C. edulis* distribution points analyzed in the present study, except for Sinaloa and southern Sonora, where no overlap was observed. The distribution of *C. edulis* quantitatively is located in the central region of the country, in an east-west strip along the Mexican volcanic axis (Figure 4). Additionally, populations of this plant are located within the environmental connectivity zone calculated in the limits of Puebla and Veracruz (Figure 4).

## 4. Discussion

The African citrus psyllid showed extensive environmental availability in Mexico, which means that this species will be able to find the environmental conditions to fulfill the stages of establishment and spreading, typical of invasive species [28]. *Tryoza erytreae* has invaded new places where it has found geographic spaces with an appropriate climate and hosts to guarantee its survival, and where it clearly separates ecologically from the Asian citrus psyllid [20,26]. In Mexico, this situation seems to be confirmed, since while the environmental conditions for *D. citri* are towards the country’s coastal areas and the lowlands of the interior [60], the ecological niche calculated for *T. erytreae* is concentrated in highland areas towards the center of the country, with few appropriate coastal zones (Figure 1).

The mean temperature of the wettest quarter seems the essential bioclimatic variable to define the environmental suitability for *T. erytrae*. This index provides mean temperatures during the wettest three months of the year, and it seems to affect species seasonal distributions probably [59]. The second most important variable is precipitation of driest month and is a useful variable if extreme precipitation conditions during the year influence the species potential range [59]. Climatic conditions are long known to affect *T. erytreae* survival: high temperatures combined with low humidity decreases eggs and first instar nymphs survival [61], preferring cooler and moister climates [25]. Several authors had mentioned the distinct climatic requirements for *T. erytrae* and *D. citri* [3,20,24,25,26,27]. Our model entirely coincides with these asseverations, projecting environmental suitability for *T. erytreae* in different, but complementary areas [60]. In fact, they point out that if the model prediction is fulfilled, all the Mexico citrus areas will have one or two of the HLB vector psyllids.

The projected ecological niche includes the main lemon producing states in the country, Michoacán, Veracruz, Oaxaca, and Colima (Figure 2 and Table 3), which represent 75% of the surface of this crop [30]. A similar situation is exact for orange (Figure 2 and Table 3), Veracruz, Tamaulipas, San Luis Potosi, and Puebla make up 77.8% of the cultivated area in Mexico [30]. Lemon and orange are two of the six main fruit trees grown in Mexico [62], and the export of citrus is one of the main economic activities of the country [63]. Therefore, the potential effects of *T. erytreae* could decrease the production capacity of infested citruses and harm the production chain; that generates in the case of lemon, up to 9 million annual wages [64], with the consequent economic and social impact [65].

Currently, there are human activities that provide these invasive species with opportunities to move beyond their geographical limits [66] and reach new spaces. In the case of Mexico, the states of Veracruz, Quintana Roo, and Michoacán have significant commercial and human affluence, in addition to appropriate environmental conditions and citrus-producing areas. This places them as the most susceptible states to be invaded by *T. erytreae*. However, it seems that *T. erytreae* could find an optimal bioclimatic route for spreading in the country through the port of Veracruz to the northwest of Puebla, and from there to central Mexico. In this space, *T. erytreae* will find cultivated and backyard citruses, since *Citrus* sp. is widely used for food, ornamental, medicinal, and so on [67]. Furthermore, *C. edulis* specimens are broadly located, providing refuge, feeding, and reproduction sites for *T. erytreae*.

*Casimiroa edulis* is a widely distributed species in Mexico and Central America, with great local appreciation. It grows wild and is cultivated in backyards for sale and consumption of its ripe fruits [68,69]. Furthermore, the fruit is used to combat insomnia [70], while the use of its seeds in hypertension treatments is widely known [71]. Characteristics of the palatability of the fruit (appearance, size, and sweet taste) and its adaptation to subtropical climates, have facilitated its dispersion and cultivation to Africa, Asia, and Oceania [72,73]. In Ethiopia and Madeira Island, *T. erytreae* affects cultivated and wild populations of *C. edulis*, with the typical symptoms of leaf galling (15, 18). The aforementioned establishes the potential capacity of *T. erytreae* to infest *C. edulis* in Mexico, using it as an alternate host to citruses in the country. Accordingly, the sampling for this psyllid should include commercial areas of fruit trees, as well as the distribution areas of this alternate species. This brings to mind the case of *Diaphorina citri* Kuwayama, also an invasive species of citrus, which was first detected in Mexico in 2009 [31] and which has *Murraya paniculata* (L.) Jack (Rutaceae) as its wild host; it is a perennial species present in urban areas where it functions as an infestation focus towards nearby citruses [74]. Currently, *D. citri* is registered in at least 368 municipalities in 21 states of the country, representing 41% of the total citrus surface nationwide [75]. With the results of this work, it is possible to recommend that the regulatory phytosanitary authorities direct monitoring and detection programs to the state of Veracruz, and thus decrease the potential impact on national citrus production and native species of the Rutaceae family.

## 5. Conclusions

The African citrus psyllid, *Tryoza erytreae*, has environmental suitability in Mexico that coincides with states of citrus production importance and with the distribution of at least one other species of Rutaceae that could function as an alternate host (*Casimiroa edulis*). The state of Veracruz proved to have bioclimatic connectivity in citrus-producing areas, and it has a seaport that offers, so far, the best conditions for the entry of *T. erytreae* to Mexico. The negative impacts associated with the transmission of pathogens by *T. erytreae* to citruses in the country can be decreased if preventive monitoring strategies prioritize citrus-producing areas and those with the presence of *C. edulis* in the state of Veracruz.

## Figures and Tables

**Table 1:** Federal entities of Mexico and cultivated surface (hectares) with four citrus species (Made with data from [30]).

State	Lemon	Orange	Tangerine	Grapefruit	Total
Veracruz	47,895.08	169,965.50	9118.90	7921.00	234,900.48
Michoacan	63,741.95	350.00	0.00	6046.00	70,137.95
Tamaulipas	7954.90	33,238.81	850.31	2196.84	44,240.86
San Luis Potosi	2033.50	32,778.59	2350.50	8.00	37,170.59
Puebla	2829.20	29,019.55	4248.80	424.05	36,521.60
Nuevo Leon	630.00	25,576.50	3607.00	1840.80	31,654.30
Oaxaca	21,500.90	4528.50	0.00	90.00	26,119.40
Colima	19,244.85	344.00	0.00	15.50	19,604.35
Yucatan	4261.83	13,163.76	996.69	721.71	19,143.99
Tabasco	7227.32	8163.50	62.00	110.00	15,562.82
Sonora	291.00	6889.00	458.00	500.50	8138.50
Guerrero	6956.79	569.08	12.00	0.50	7538.37
Jalisco	6557.97	609.25	0.00	60.00	7227.22
Hidalgo	279.60	5762.90	30.00	0.00	6072.50
Campeche	2099.30	2577.70	70.00	652.50	5399.50
Chiapas	2814.85	1905.85	51.90	0.00	4772.60
Quintana Roo	2375.50	1473.00	20.00	0.00	3868.50
Sinaloa	1293.20	1626.00	55.00	95.00	3069.20
Baja California Sur	57.00	2959.55	4.00	20.15	3040.70
Nayarit	2850.81	77.66	3.25	0.00	2931.72
Durango	255.81	283.60	0.00	199.00	738.41
Zacatecas	625.50	0.00	0.00	0.00	625.50
Morelos	399.50	186.40	8.50	4.00	598.40
Baja California	126.55	274.00	2.50	13.20	416.25
Estado de Mexico	133.50	26.00	5.00	0.00	164.50
Guanajuato	86.00	0.00	0.00	0.00	86.00
Aguascalientes	57.20	3.00	0.00	0.00	60.20

**Table 2:** Biolimatic variables incorporated in the *Tryoza erytreae* (Hemiptera: Triozidae) ecological niche modelling in Mexico [47,59].

Variable	Bioclimatic Predictor	% Contribution to Explain the Model
BIO1	Annual mean temperature	0.5
BIO2	Mean diurnal range	0.8
BIO3	Isothermality	1.9
BIO4	Temperature seasonality	NA
BIO5	Maximum temperature of warmest month	NA
BIO6	Minimum temperature of coldest month	NA
BIO7	Temperature annual range	NA
BIO8	Mean temperature of wettest quarter	69.0
BIO9	Mean temperature of driest quarter	NA
BIO10	Mean temperature of warmest quarter	NA
BIO11	Mean temperature of coldest quarter	NA
BIO12	Annual precipitation	NA
BIO13	Precipitation of wettest month	NA
BIO14	Precipitation of driest month	25.3
BIO15	Precipitation seasonality (coefficient of variation)	0.5
BIO16	Precipitation of wettest quarter	NA
BIO17	Precipitation of driest quarter	NA
BIO18	Precipitation of warmest quarter	2.1
BIO19	Precipitation of coldest quarter	NA

NA = bioclimatic variable without contribution for model explanation. A Spearman correlation with all the bioclimatic variables was conducted in Past 2.17c software, and were eliminated those variables with higher correlation values of 0.75 and −0.75.

**Table 3:** Level of risk of *Trioza erytreae* (Hemiptera: Triozidae) invasion of 27 citrus-producing states in Mexico, according to the percentage of the surface where environmental availability is projected.

>60% of Environmental Availability, States with More Than 10 Thousand Cultivated Hectares	Risk	30–59% Environmental Availability, States with 1–10 Thousand Hectares Cultivated	Risk	<29% Environmental Availability, States with Less Than a Thousand Hectares Cultivated	Risk
Veracruz Michoacan Tamaulipas San Luis Potosi Puebla Nuevo Leon Oaxaca Colima Yucatan Tabasco	1 2 2 1 1 2 1 3 3 1	Sonora Guerrero Jalisco Hidalgo Campeche Chiapas Quintana Roo Sinaloa Baja California Sur Nayarit	3 3 1 1 3 2 2 3 3 3	Durango Zacatecas Morelos Baja California Mexico Guanajuato Aguascalientes	1 1 3 3 1 1 1

1 = high risk; 2 = medium risk; 3 = low risk.

## Data Availability

Not applicable.
